# Supplementary material for: Input-dependent modulation of MEG gamma oscillations reflects gain control in the visual cortex
Source: Sci Rep. 2018 May 31;8:8451. doi: 10.1038/s41598-018-26779-6 (PMC5981429; doi:10.1038/s41598-018-26779-6)
Supplement: Supplementary file 1 — Supplementary information [file 41598_2018_26779_MOESM1_ESM.docx]

**Supplementary information**

**Input-dependent modulation of MEG gamma oscillations reflects gain control in the visual cortex.**

Elena V. Orekhova^1,2^, Olga V. Sysoeva^2^, Justin F.Schneiderman^3,4^, Sebastian Lundström^1^, Ilia A. Galuta^5^, Dzerasa E. Goiaeva^5^, Andrey O. Prokofyev^2^, BushraRiaz^3,4^, Courtney Keeler^3^, Nouchine Hadjikhani^1,6^, Christopher Gillberg^1^ , Tatiana A. Stroganova^2,5^

1. University of Gothenburg, Gillberg Neuropsychiatry Centre (GNC), Gothenburg, Sweden;

2.Center for Neurocognitive Research (MEG Center), Moscow State University of Psychology and Education;

3. MedTech West, Gothenburg, Sweden;

4. University of Gothenburg, Institute of Neuroscience& Physiology, Gothenburg, Sweden;

5. Autism Research Laboratory, Moscow State University of Psychology and Education, Moscow, Russia;

6. Harvard Medical School, MGH/MIT/HST MartinosCenter for Biomedical Imaging, Charlestown, MA USA

**Corresponding author:**

Elena V Orekhova,

Center for Neurocognitive Research (MEG Center),

Moscow State University of Psychology and Education,

Moscow, Russia;

[orekhova.elena.v@gmail.com](mailto:orekhova.elena.v@gmail.com)

**Supplementary Methods**

***Stimuli presentation***

The subjects sat in a dim magnetically shielded room, with their head resting against the back of and positioned as closely as possible to the top of the helmet-shaped surface of the helium dewar. For adults in experiment 1 and for children in experiment 2 we used a PT-D7700E-K DLP projector that presented images with 1280 х 1024 screen resolution and 60 Hz refresh rate. For adults in experiment 2 the stimuli were presented using FL35 LED DLP projector with 1920 х 1080 screen resolution and 120 Hz refresh rate. The default gamma correction was applied for the both projectors.

***Details on experimental task***

To keep participants alert we asked them to respond to the change in the stimulation flow (stop of the motion in both experiments or disappearance of the static stimulus in experiment 1) with a button press. The response period was individually adjusted in children (see^1^ for details) and was fixed at 1000 ms in adults. If no response occurred within the response period, the grating was substituted by a discouraging message “too late!” (in Russian/Swedish for participants in Moscow vs Gothenburg) that remained on the screen for 2000 ms, after which a new trial begun. The participants were instructed to constantly maintain their gaze at the fixation cross in the intervals between the stimuli.

Individual omission errors and commission errors (responses that occurred during motion or earlier than 150 ms following termination of motion) were measured in all participants except two children for whom the behavioral responses were not recorded due to equipment error. Video monitoring has shown that these two children watched the stimuli and performed the task (pressed the button). Therefore, their data were not excluded from the analysis. In all other cases the error trials were excluded from the MEG analysis.

Static stimulus and those drifting at different velocities were intermixed and appeared in a random order within each of three experimental blocks. The participants responded with either the right or the left hand (50% trials each) in a sequence that was counterbalanced between blocks and participants. Each of the four (in experiment 1) or three (in experiment 2) stimuli types was presented 30 times within each experimental block.

***Details on data recording***

In adults in experiment 1 and in children in experiment 2, neuro-magnetic brain activity was recorded at the Moscow MEG Centre (the Moscow State University of Psychology and Education) using a 306-channel detector array (Vectorview; Neuromag, Helsinki, Finland) positioned in a magnetically shielded room (AK3b; Vacuumschmelze, Hanau, Germany). In adults in experiment 2, MEG was recorded at the NatMEG Centre (The Swedish National Facility for Magnetoencephalography, KarolinskaInstitutet, Stockholm) using a similar 306-channel system (ElektaNeuromag TRIUX) located in 2-layer magnetically shielded room (Vacuumschmelze GmbH). Both systems comprise 102 identical triple sensor elements. Each sensor element consists of three superconducting quantum interference devices, two with orthogonal planar gradiometer pickup coils and one with a magnetometer pickup coil configuration. Four electrooculogram (EOG) electrodes were used to record horizontal and vertical eye movements. EOG electrodes were placed at the outer canti of the eyes and above and below the left eye. To monitor the heartbeats at the Moscow MEG centre one ECG electrode was placed at the manubrium sterni and the other one at the mid-axillary line (V6 ECG lead). At the Karolinska MEG cenre the ECG electrodes were placed at left and right sides of the chest under the collarbone. MEG, EOG, and ECG signals were recorded with a band-pass filter of 0.03–330 Hz in Moscow and 0.1-330 Hz in Stockholm, digitized at 1000 Hz, and stored for off-line analysis.

***MEG data preprocessing***

The data was first de-noised using the Temporal Signal-Space Separation (tSSS) method^2^ implemented in MaxFilter™ (v2.2) with parameters: ‘-st’=4 and ‘-corr’=0.90. For all three experimental blocks, the head origin position was adjusted to the initial head origin position in the block #2. For further pre-processing we used the MNE-python toolbox^3^ as well as custom Python and Matlab scripts.

To remove biological artifacts (blinks, heart beats, and in some cases myogenic activity), we then applied independent component analysis (ICA). The MEG periods with too high (4000e-13 fT/cm for gradiometers and 4e-12 fT for magnetometers in adults, 4000e-13 fT/cm for gradiometers and 8e-12 fT for magnetometers in children) or too low (0.1e-12 fT/cm for gradiometers and 1e-13 fT for magnetometers) amplitudes were excluded from the analysis. The number of independent components was set at dimensionality of the raw ‘SSS’ed’ data (usually around 70). We further used an automated MNE-python procedure to detect artificial EOG and ECG components, which we complemented with visual inspection of the ICA results. The number of rejected artifact components was usually 1 for vertical eye movements, 0-3 for cardiac and 0-6 for myogenic artifacts.

The epochs containing strong muscle artifacts were excluded by thresholding the mean absolute value of the high-frequency (>70 Hz) signal. The threshold was set at 3 standard deviations from the power of the 70-Hz high-passed signal averaged across channels. The epochs left were visually inspected for the presence of undetected high-amplitude bursts of myogenic activity and those contaminated by such artifacts were manually marked and excluded from the analysis.

***Time-frequency analysis of the MEG data at the sensor level***

To decrease the contribution of phase-locked activity related to appearance of the stimulus on the screen, as well as photic driving that could be induced by the temporal frequency of the stimulation and screen refresh rate or by their interaction, we subtracted averaged evoked responses from each single data epoch using ‘subtract_evoked’ MNE function.

The gamma response strength was estimated in dB in 200-1200 ms post-stimulus period relative to the -900 to 0 ms period of pre-stimulus baseline (the 400 ms windows were centered at -700: 50: -200 ms time points for the pre-stimulus interval and at 400:50:1000 ms points for post-stimulus interval).

To identify the location of the ‘maximal response sensor pair’ the power changes were averaged over the two gradiometers of each sensors triplet, over the 400-1000 ms post-stimulus period and over frequencies of the gamma range (50-110 Hz in children and 35-100 Hz in adults). Because our previous study showed that detectable gamma response on the group level is observed at posterior gradiometer sensors (with the maximum at the 'MEG2112/3' pair of gradiometers), the location of the maximal increase in gamma power was defined at one of the selected posterior locations ('MEG1932/3', 'MEG1922/3', 'MEG2042/3', 'MEG2032/3', 'MEG2112/3', 'MEG2122/3', 'MEG2342/3' and 'MEG2332/3'), separately for each condition.

***Time-frequency analysis of the MEG data at the source level***

The structural MRIs (1 mm^3^ T1-weighted) were available for 23 adult participants in experiment 2. For these participants we performed source analysis using FieldTrip open source software (<http://www.ru.nl/neuroimaging/fieldtrip/>). We sought to check for the similarity between gamma response parameters measured at the source and the sensor levels, as well as to assess position of the most significant gamma increase (‘maximally induced voxel’) in each velocity conditions. Prior to analysis each subject’s brain has been morphed to the MNI template brain using linear normalization and 0.6 mm grid. To perform source localization we then adapted the two-step approach. As the first step time-frequency analysis was performed on the artifact-free sensor data epochs (-0.9 to 0 pre-stimulus and 0.2 to 1.2 post-stimulus) using multitaper method. The analysis window was centered at the sensor-defined subject/condition specific frequency ±25 Hz. The DICS inverse-solution algorithm^4^ was used to derive the common source spatial filters. Subsequently, bootstrap resampling source statistics was performed (with 10000 Monte Carlo repetitions) to verify for each participant and condition presence of a significant (p<0.05) brain cluster of post-stimulus increase in gamma power in the visual cortex (L/R cuneus, lingual, occipital superior, middle occipital, inferior occipital, or calcarine areas according to the AAL atlas^5^ ).

At the second step the signal was filtered in 30-120 Hz range, the ‘virtual sensors' time series were extracted for each of 6855 brain voxels, and the time-frequency analysis (multitaper method, ±5 Hz smoothing, ~1Hz frequency resolution) has been performed. Then, the gamma response parameters were computed for its spatial maximum, which was defined as 26 voxels adjacent to and including one exhibiting the most significant post-stimulus increase in 45-90 Hz power. To assess the source-derived gamma response frequency and amplitude we calculated post-/pre-stimulus ratio for each frequency in the broader high-frequency range (30-120 Hz) and then assessed weighted subject/condition specific frequency and amplitude using the same approach that was applied in the sensor space analysis.

**References**

1 Orekhova, E. V. *et al.* Frequency of gamma oscillations in humans is modulated by velocity of visual motion. *J Neurophysiol* **114**, 244-255, doi:10.1152/jn.00232.2015 (2015).

2 Taulu, S. & Hari, R. Removal of magnetoencephalographic artifacts with temporal signal-space separation: demonstration with single-trial auditory-evoked responses. *Hum Brain Mapp* **30**, 1524-1534, doi:10.1002/hbm.20627 (2009).

3 Gramfort, A. *et al.* MEG and EEG data analysis with MNE-Python. *Front Neurosci* **7** (2013).

4 Gross, J. *et al.* Dynamic imaging of coherent sources: Studying neural interactions in the human brain. *P Natl Acad Sci USA* **98**, 694-699, doi:DOI 10.1073/pnas.98.2.694 (2001).

5 Tzourio-Mazoyer, N. *et al.* Automated anatomical labeling of activations in SPM using a macroscopic anatomical parcellation of the MNI MRI single-subject brain. *Neuroimage* **15**, 273-289, doi:10.1006/nimg.2001.0978 (2002).

**Supplementary Results**

**Results of the source analysis of gamma oscillations during stimulating with high-contrast gratings moving with ‘slow’ (1.2°/s), ‘medium’ (3.6°/s) and ‘high’ (6.0°/s) velocities.**

In each of 23 participants the DICS beamformer analysis revealed a significant cluster of voxels in the visual cortex that demonstrated gamma increase in response to the ‘slow’ and ‘medium’ velocity. In response to the ‘fast’ velocity, a significant cluster was present in 18 of 23 participants. In all three conditions, the most frequent location of the ‘maximally induced voxel’ was in the calcarine sulcus (in 17/23 subjects in the ‘slow’ condition, in 16/23 subjects in the ‘medium’ condition and in 11/18 subjects in the ‘fast’ condition, see the *Supplementary table 1* for the MNI coordinates of the ‘maximally induced voxels’). Figure 2C illustrates source distribution of the gamma response obtained using DICS beamforming technique. No systematic shifts of the ‘maximally induced voxel’ in either x, y or z coordinate has been observed between the three velocity condition (rmANOVAs: all p’s>0.4). The mean intra-individual distances between ‘maximally induced voxels’ were 5.4 mm (sd=6.2) for the ‘slow’ vs ‘medium’ conditions, 10 mm (sd=7.6) for the ‘medium’ vs ‘fast’ conditions, and 9.3 mm (sd=8.5) for the ‘slow’ vs ‘fast’ conditions. The group average [x y z] position of the ‘maximally induced voxel’ in the MNI coordinates was [0.0 -93.7 -0.7] for the ‘slow’ condition, [-0.6 -94.0 -0.7] for the medium condition and [2 -94.7 0.0] for the fast condition. Considering 6 mm spatial resolution of the grid, the mean position of the gamma response maximum corresponded to the same MNI template voxel in the left calcarine sulcus in all velocity conditions.

***Supplementary table 1*. Location of the ‘maximally induced voxel’ in the ‘slow’, ‘medium’ and ‘fast’ velocity conditions in each of 23 adult participants in whom the source localization has been performed. The results are presented only in case of significant gamma cluster in the visual areas.**

|  | Location of the ‘maximally induced voxel’ | | | MPI coordinates of the ‘maximally induced voxel’ | | | | | | | | | |
| --- | --- | --- | --- | --- | --- | --- | --- | --- | --- | --- | --- | --- | --- |
| Subj | Slow | Medium | Fast | Slow | | | Medium | | | Fast | | | |
|  |  |  |  | x1 | y1 | z1 | x2 | y2 | z2 | | x3 | y3 | z3 |
| 1 | Calcarine R | Calcarine L | Calcarine R | 20 | -100 | 2 | -4 | -100 | 8 | | 14 | -100 | -4 |
| 2 | Calcarine L | Calcarine L | Calcarine L | 2 | -94 | 2 | 2 | -94 | -4 | | 2 | -94 | 8 |
| 3 | Lingual L | Lingual L | Lingual L | -16 | -88 | -10 | -10 | -88 | -10 | | -10 | -88 | -10 |
| 4 | Lingual L | Lingual L | Lingual L | -10 | -88 | -16 | -10 | -88 | -16 | | -10 | -100 | -16 |
| 5 | Occipital Sup L | Occipital Mid L | Occipital Mid L | -10 | -94 | 2 | -16 | -94 | 2 | | -22 | -100 | 8 |
| 6 | Calcarine L | Calcarine L | Calcarine L | -4 | -94 | 2 | 2 | -100 | 2 | | -4 | -100 | 8 |
| 7 | Calcarine L | Calcarine L | Calcarine L | -16 | -94 | -4 | -16 | -94 | -4 | | 2 | -94 | 2 |
| 8 | Calcarine L | Calcarine L |  | -4 | -94 | -4 | -4 | -94 | 2 | |  |  |  |
| 9 | Calcarine L | Calcarine R | Calcarine R | 2 | -94 | 2 | 8 | -94 | 2 | | 8 | -94 | 2 |
| 10 | Calcarine R | Calcarine L | Calcarine R | 8 | -94 | 2 | 2 | -94 | 2 | | 8 | -94 | 2 |
| 11 | Occipital Mid L | Calcarine L |  | -10 | -100 | 2 | -4 | -106 | -4 | |  |  |  |
| 12 | Calcarine R | Calcarine R | Lingual R | 14 | -100 | -4 | 14 | -100 | -4 | | 14 | -100 | -10 |
| 13 | Calcarine L | Calcarine L | Calcarine L | 2 | -94 | 2 | 2 | -94 | 2 | | 2 | -94 | 2 |
| 14 | Calcarine L | Lingual R | Lingual R | -4 | -82 | -10 | 8 | -82 | -10 | | 8 | -82 | -10 |
| 15 | Calcarine R | Calcarine L |  | 8 | -82 | 8 | 2 | -94 | -4 | |  |  |  |
| 16 | Calcarine L | Calcarine L |  | -4 | -100 | 2 | -4 | -94 | -10 | |  |  |  |
| 17 | Calcarine L | Calcarine L | Occipital Mid L | 2 | -100 | 2 | 2 | -100 | 2 | | -10 | -106 | 2 |
| 18 | Cuneus L | Cuneus L | Calcarine R | -4 | -94 | 14 | -4 | -94 | 14 | | 8 | -94 | 8 |
| 19 | Calcarine L | Calcarine L |  | 2 | -88 | -4 | 2 | -88 | -4 | |  |  |  |
| 20 | Calcarine R | Calcarine R | Calcarine R | 8 | -94 | 2 | 14 | -100 | 2 | | 8 | -94 | 2 |
| 21 | Calcarine L | Calcarine L | Calcarine L | 2 | -88 | 2 | -4 | -88 | 2 | | -4 | -88 | 2 |
| 22 | Occipital Mid L | Occipital Mid L | Cuneus R | -10 | -100 | 2 | -10 | -100 | 2 | | 20 | -94 | 8 |
| 23 | Calcarine R | Lingual R | Calcarine L | 14 | -94 | -4 | 8 | -88 | -4 | | 2 | -88 | -4 |
